# Kinematic Profile of Visually Impaired Football Players During Specific Sports Actions

**DOI:** 10.1038/s41598-019-47162-z

**Published:** 2019-07-23

**Authors:** Sara Finocchietti, Monica Gori, Anderson Souza Oliveira

**Affiliations:** 1 U-VIP: Unit for Visually Impaired People, Fondazione Istituto Italiano di Tecnologia, Genova, Italy; 2 Department of Materials and Production, Aalborg University, Aalborg, Denmark

**Keywords:** Neurophysiology, Disability

## Abstract

Blind football, or Football 5-a-side, is a very popular sport amongst visually impaired individuals (VI) worldwide. However, little is known regarding the movement patterns these players perform in sports actions. Therefore, the aim of this study was to determine whether visually impaired players present changes in their movement patterns in specific functional tasks compared with sighted amateur football players. Six VI and eight sighted amateur football players performed two functional tasks: (1) 5 m shuttle test and (2) 60 s ball passing against a wall. The sighted players performed the tests while fully sighted (SIG) as well as blindfolded (BFO). During both tasks, full-body kinematics was recorded using an inertial motion capture system. The maximal center-of-mass speed and turning center-of-mass speed were computed during the 5 m shuttle test. Foot resultant speed, bilateral arm speed, and trunk flexion were measured during the 60 s ball passing test. The results showed that VI players achieved lower maximal and turning speed compared to SIG players (p < 0.05), but BFO were slower than the VI players. The VI players presented similar foot contact speed during passes when compared to SIG, but they presented greater arm movement speed (p < 0.05) compared to both SIG and BFO. In addition, VI players presented greater trunk flexion angles while passing when compared to both SIG and BFO (p < 0.05). It is concluded that VI players present slower speed while running and turning, and they adopt specific adaptations from arm movements and trunk flexion to perform passes.

## Introduction

Football is the most practiced and followed sport in the world, in which players need to efficiently and effectively execute the skilled movement, applying cognitive, perceptual and motor skills in ever-changing gaming contexts^1^. Blind football (officially called Football 5-a-side) is currently a Paralympic sport that is a variation of futsal, designed for players who are visually impaired (VI)^2^. Players are assigned to one of three sport classes based on their level of visual impairment^3^: (1) B1 - totally blind; from no light perception up to light perception but inability to recognize the shape of a hand; (2) B2 - partially sighted; able to recognize the shape of a hand up to a visual acuity of 2/60 or a visual field of less than 5 degrees; (3) B3 - partially sighted; visual acuity from 2/60 to 6/60 or visual field from 5 to 20 degrees. It is a five against five games in a field measuring 40 m × 20 m. In blind football, the football contains ball bearings that rattle and make the ball’s location accessible for VI players through auditory stimuli^4^. Players call out “yeah” and their names to make teammates aware of their presence. As a result, spectators must remain silent whilst watching the game until a goal is scored. The goalkeeper is sighted or partially sighted, to allow for the guidance of the other players who wear eyeshades to account for differences in blindness severity^3^. Blind football is quite popular worldwide, having organized national leagues in France, Brazil, and England.

The physical fitness of football athletes has been dramatically improved in the last decades, as players are able to run faster and farther during the matches^5^. Some of these advances were achieved by the use of biomechanical analysis that describes the player’s motion. Understanding movement patterns have been essential for coaches and athletes, as it allows proposing changes to these patterns to improve performance^6,7^. Despite the considerable popularity of blind football, there is limited information regarding movement patterns of VI players. It has been shown that VI goalball and football athletes have similar self-selected walking speed, but lower static balance, when compared to sighted individuals^8^. In addition, these authors showed that VI players presented a greater fear of falling during sports practices. Therefore, evaluating movement patterns of VI football players in specific sports actions can be valuable to describe their disability-related movement limitations. Subsequently, this information can help in designing novel training methods to maximize the performance of blind football players and playing experiences.

It is widely believed that blind individuals are better than sighted in the audio skills but this is not always true and recent results show that in some cases they have big impairments in audio spatial skills^9–11^. During football, the lack of visual input for blind players changes the way they perceive the ball’s location for a kick or a pass, likely evoking greater participation from the auditory system and overall postural control through somatosensory information to maintain postural control with no visual inputs^12^. Therefore, it is essential to assess the movement patterns of VI players in the most natural conditions possible. Inertial motion capture systems (IMC) have become highly popular in recent years, providing acceptable measurements of human kinematics in different movement conditions^13,14^. Especially for sports activities, IMCs allows recordings of kinematic data in more natural conditions, such as open spaces like football courts. This feature from IMCs is highly suitable to record kinematic profiles of VI football players while they perform football movements.

To date, there are no studies investigating the movement patterns of blind players during game situations. Therefore, the aim of this study was to determine whether visually impaired players present changes in their movement patterns in specific functional tasks when compared to sighted amateur football players. It was hypothesized that visually impaired players would run slower, take more time to perform turns and perform less correct passes than sighted players. Moreover, visually impaired players will present distinct kinematic patterns when compared to sighted players. In addition, we hypothesized that blindfolded players would be slower than visually impaired players and assume changes in body posture to being able to perform simple passes. The results of this study can contribute to increasing our understanding of the motor performance of VI individuals.

## Methods

### Participants

Six male visually impaired (VI, 2 blind, age range: 25–38 years) and eight age-matched healthy controls participated in the study. All participants were males and amateur players (age range: 26–40), practicing football 1–2 times per week and participating at the National Italian Blind Football league. The vision loss of the early blind had different etiology. One player was born blind whereas another lost his vision at the age of four, as indicated in Table 1. The healthy controls were amateur players that practice both football and five-a-side football 1–2 times per week. Both VI and sighted individuals have practiced football for at least 10 years. Written informed consent was obtained from each subject prior to inclusion in the study. The study was conducted in accordance with the Declaration of Helsinki and approved by the local ethics committee (ASL3 Genovese, Italy).

**Table 1:** Age and visual impairment characteristics of the visually impaired players participating in this study.

	age	Etiology	Residual vision	Age of complete blindness
P1	38	Retinitis pigmentosa	None	20
P2	32	Retinitis pigmentosa	Light and shadows	25
P3	18	Leber amaurosis, nistagmus	1/20	/
P4	25	Retinitis pigmentosa	Lights and shadows	17
P5	48	Congenital Glaucoma	None	6

### Experimental design

In a single session, participants performed two functional tasks in a gymnasium containing an official futsal court: 5 m shuttle and ball pass against a wall. The control group performed the tasks at first without vision (blindfolded, BFO), and then with vision (SIG) so that the sighted blindfolded players could not know in advance the football area. Kinematic data were acquired using an inertial motion capture system, and the horizontal center-of-mass speed was extracted to describe the maximum speed and turning speed during the shuttle test. The number of passes, foot, and arms speed while passing, as well as the trunk flexion angle at the T8/T9 vertebrae level, were computed from the ball passing test.

### Familiarization to blindfolded conditions

Following the appropriate placement of the inertial motion capture suit, all sighted participants were blindfolded and asked to familiarize to the environment (e.g., the court and the general sounds from their surroundings). Initially, all participants walked and ran throughout the court for approximately 10 minutes, familiarizing to the court limits and to moving without visual feedback, just following the voice commands from one experimenter. In addition, blind football handling and passing were introduced to the BFO participants. Each participant was familiarized to the sounds of the ball, and the timing required to decode when the ball was approaching them, as well as trying to pass the ball back to the experimenter, being guided auditory clues. The familiarization to the blindfolded condition was considered successful when the participant felt comfortable to perform running, passing and changing directions following auditory clues. Additional time was allowed if a participant required more time to familiarize to restricted vision.

### The 5 m shuttle and ball passing tests

For the 5 m shuttle test (adapted from Boddington and co-workers^15^), participants were asked to perform a 10 m shuttle test by running in a 5 m track marked on the floor and back to the original position. The trial was considered successful if both feet have crossed the 5 m line while turning. For VI players, one experimenter was positioned parallel to the 5 m line and whistled when the participant was with the trunk over the 5 m line, which indicated that he could turn and run back. Moreover, this auditory signal minimized the possibility of non-straight running after turning. A total of 5 successful trials were recorded for each participant, and average across trials was computed for the maximum speed and turning speed for further statistical analysis.

Regarding ball passes against a wall, participants were asked to perform passes at the floor level against a wall located 5 m in front of them. This wall was 10 m wide and the test started with the ball positioned at the central position. A preliminary study on 11 healthy, young and sighted recreational football players has shown a high intra-class correlation coefficient across three different test days (r = 0.995, see Supplementary Table 1). Participants were instructed to perform as many passes as possible for 60 seconds while keeping an approximate distance of 5 m from the wall. The test was conducted using an official blind football which contains rings embedded, therefore VI and BFO participants could hear the location of the ball to perform the passes.

### The inertial motion capture system

An IMC (Xsens MVN Link, Xsens Technologies BV, Enschede, The Netherlands) and its respective software (Xsens MVN Studio version 4.2.4, Enschede, The Netherlands) were used to record full-body kinematics at a sampling rate of 240 Hz. The IMC consisted of 17 inertial measurement unit modules (25 × 35 × 8 mm, 30 g) mounted on a tight-fitting Lycra suit containing pre-defined locations for sensor placement. The IMUs were placed bilaterally in the following locations: shoulder, arm, forearm, hand, thigh, shank, and foot. In addition, IMUs were placed on the head (using a headband), on the chest and on the sacrum. The manufacturer’s sensor calibration procedure was followed by asking participants to assume different body poses such as N-pose (quiet standing with arms alongside the body) and T-pose (quiet standing with arms abducted 90° and horizontally aligned in the frontal plane). This calibration procedure assured the different IMUs were correctly representing the body’s segments in the three-dimensional space^16^. The manufacturer’s recommendations to avoid sources of electromagnetic fields were followed to assure the quality of the acquired data.

### Data processing

The orientation of each inertial measurement units was obtained by fusing accelerometer, gyroscope and magnetometer signals using an extended Kalman filter embedded in the IMC recording software^17^. The IMC software computed the three-dimensional position vectors for all sensors. The software subsequently computed automatically the center-of-mass position from each body segment, as well as the full-body center-of-mass (COM) from these position vectors. Moreover, the IMC software partitioned the trunk kinematic data into four different segments (L3, L5, T8, and T12 vertebrae), and generated joint angles for upper and lower limbs, as well as for trunk spinal joints.

In this study, we focused on the displacement of the full-body COM, kicking foot as well as the ipsilateral and contralateral arms. In addition, we investigated trunk kinematics during ball passes through the flexion angle for the lumbar (L1/T12), thoracic (T9/T8) and cervical trunk levels (T1/C7). All data from position vectors and joint angles were low-pass filtered (6 Hz, second-order Butterworth zero-phase). The COM, foot and arm segments position vectors were derived to generate velocity vectors. The resultant trunk speed was subsequently defined as:

$$S(i)=\sqrt{x{(i)}^{2}+y{(i)}^{2}+z{(i)}^{2}}$$

where for each time frame (*i*), *S* was the resultant speed from the velocity vectors in the anterior-posterior (*x*), medial-lateral (*y*) and vertical directions (*z*). Data were analyzed using custom scripts programmed in MATLAB^®^ (R2015b, Mathworks Inc., Natick, MA USA).

### Data analysis – 5-m shuttle test

**Figure 1:**
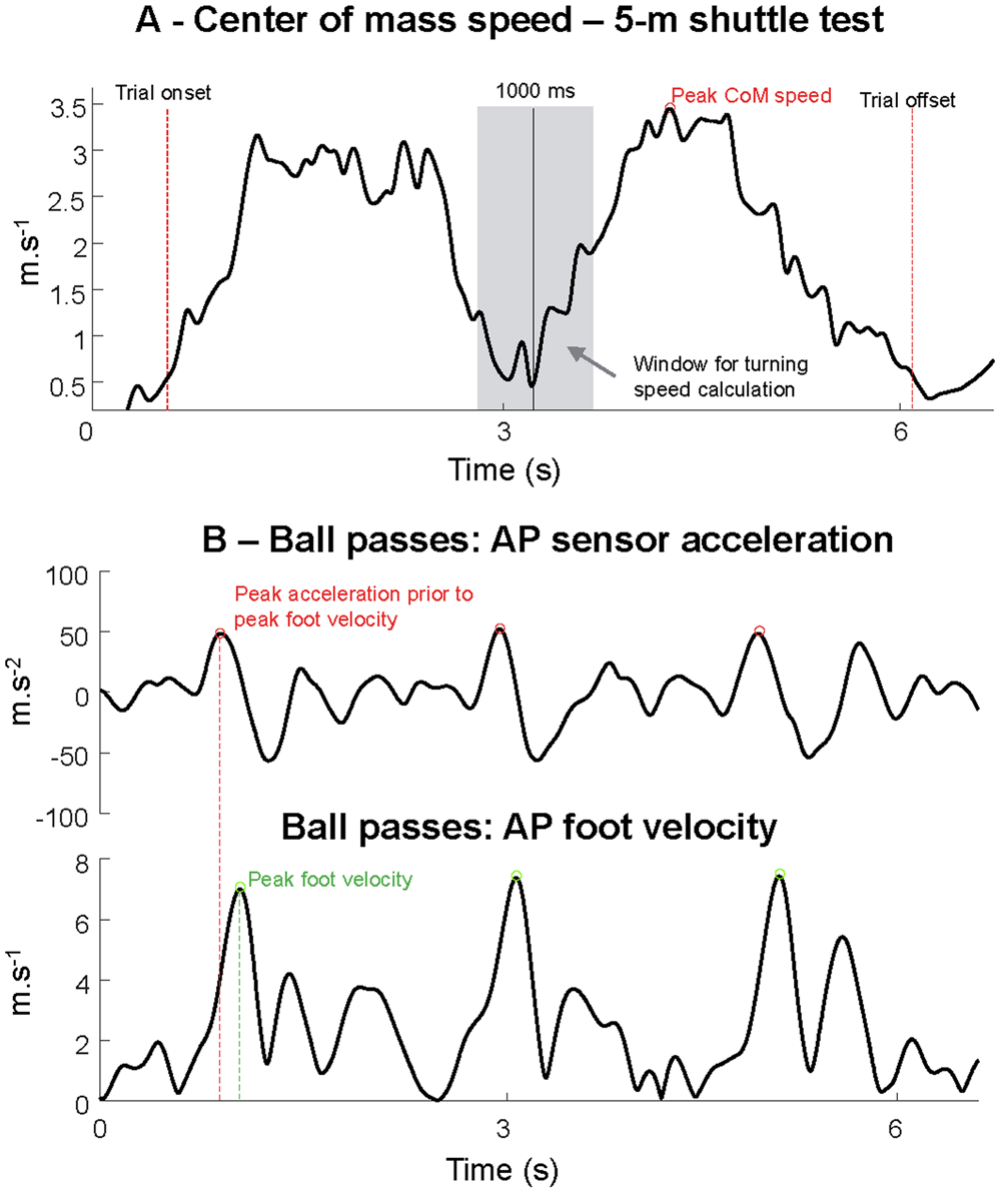
Illustration of the center of mass (CoM) resultant speed during the 5-m shuttle run test (**A**, *Top panel*) used to compute average and maximal running speed, as well as turning speed (*defined during the gray shaded area*). In (**B**) (*bottom panel*) the use of anterior-posterior (AP) foot sensor acceleration and AP foot speed to define peak AP foot speed and subsequent foot acceleration.

From the kinematic data, the shuttle period was defined from the period where the COM resultant speed was greater than 0.25 m/s. The number of strides for the dominant leg was defined from the dominant foot displacement in the shuttle running direction. The maximum speed was defined as the maximum resultant speed achieved throughout the test (Fig. 1A). In addition, we defined the turning period from −500 to 500 ms around the instant where the COM position was the farthest from the origin in the shuttle running direction. The average COM resultant speed during this turning period was defined as the COM turning speed.

### Data analysis – ball passes

The instants of foot contact to the ball were defined as the peak horizontal foot acceleration throughout the 60-second recordings (Fig. 1B), followed by visual inspection of the time indexes using the graphical representation of the participant’s task in the recording software. The resultant foot speed at the moment of contact was found using the time indexes. The resultant trunk speed was defined from 0 to 1000 ms around foot contact to the ball. In addition, the resultant speed of the ipsilateral and contralateral arms was defined from −250 to 250 ms around foot contact to the ball. Finally, the trunk flexion angle data from L1/T12, T9/T8, and T1/C7 were averaged within −250 to 250 ms around foot contact to the ball, to describe the trunk flexion during the passes.

### Statistical analysis

The Statistical Package for the Social Sciences (IBM SPSS Inc. Version 23.0, Chicago, IL, USA) was used for statistical analysis. The normality of the dependent variables (resultant speed and joint angles) was assessed using Shapiro-Wilk tests, where both variables demonstrated normal distribution (p > 0.05). The differences across the three different groups (VI *vs* BFO *vs* SIG) for each variable were assessed using ANOVA 1-way, followed by Bonferroni post-hoc tests when necessary. The significance level was set at <0.05. partial eta-squared values are reported (ŋp^2^).

## Results

### The 5-m shuttle test

**Figure 2:**
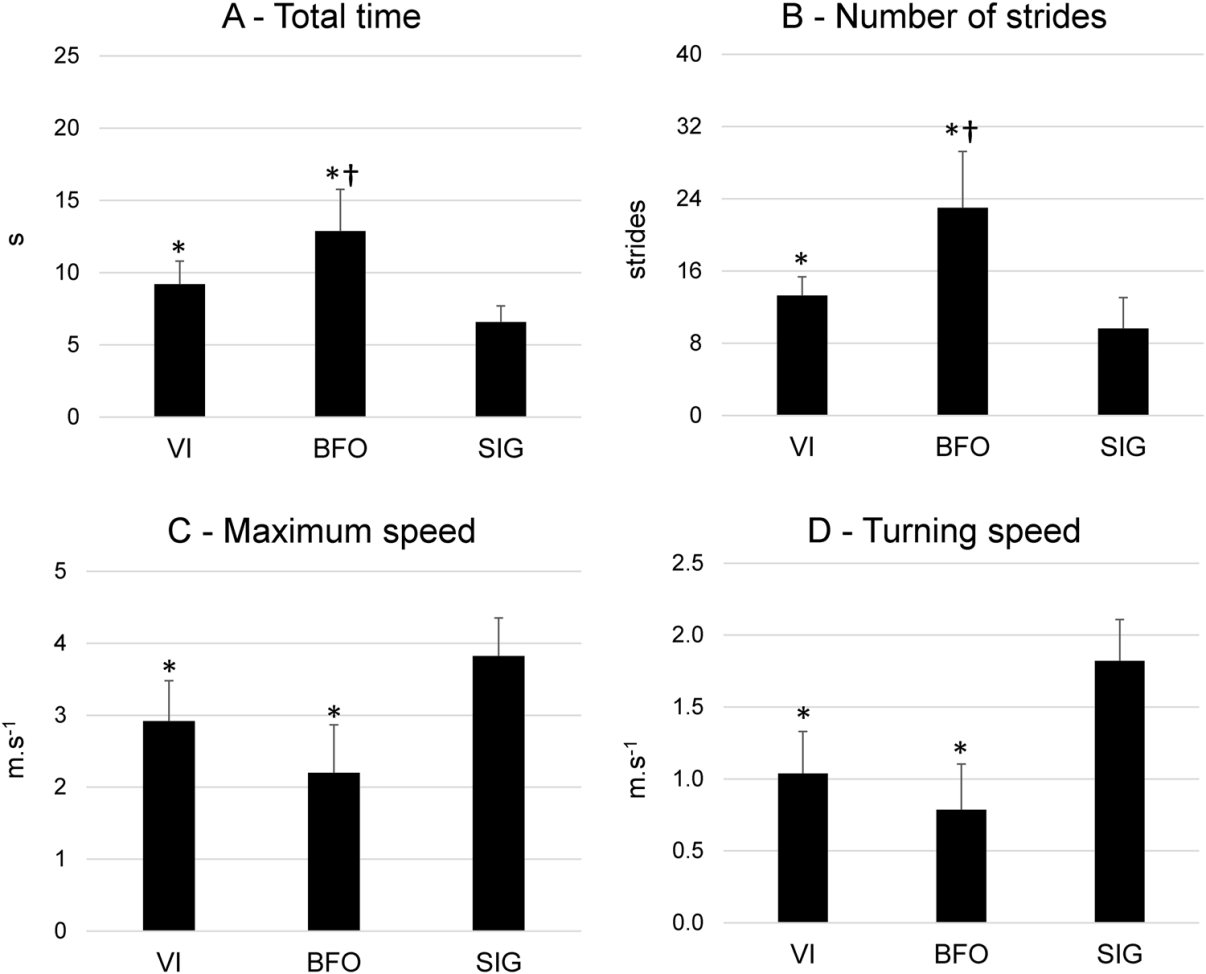
Mean (SD) of total time (**A**), number of strides (**B**), maximum speed (**C**) and turning speed (**D**) during the 5-m shuttle run test for visually impaired individuals (VI), sighted blindfolded (BFO) and sighted individuals (SIG). *Denotes significant differences in relation to SIG (p < 0.05); ^†^denotes a significant difference in relation to VI (p < 0.05).

The SIG group was significantly faster and performed fewer stride cycles during the shuttle test in comparison to VI and BFO (p = 0.00001, ŋp^2^ = 0.67, Fig. 2A,B), whereas VI was faster and performed fewer strides than BFO (p = 0.00005, ŋp^2^ = 0.67). The maximum speed (Fig. 2C) and the turning speed (Fig. 2D) were comparable between VI and BFO, while SIG ran at the highest speed, and at the fastest turning speed (p = 0.0002, ŋp^2^ = 0.63).

### Ball passes test

**Figure 3:**
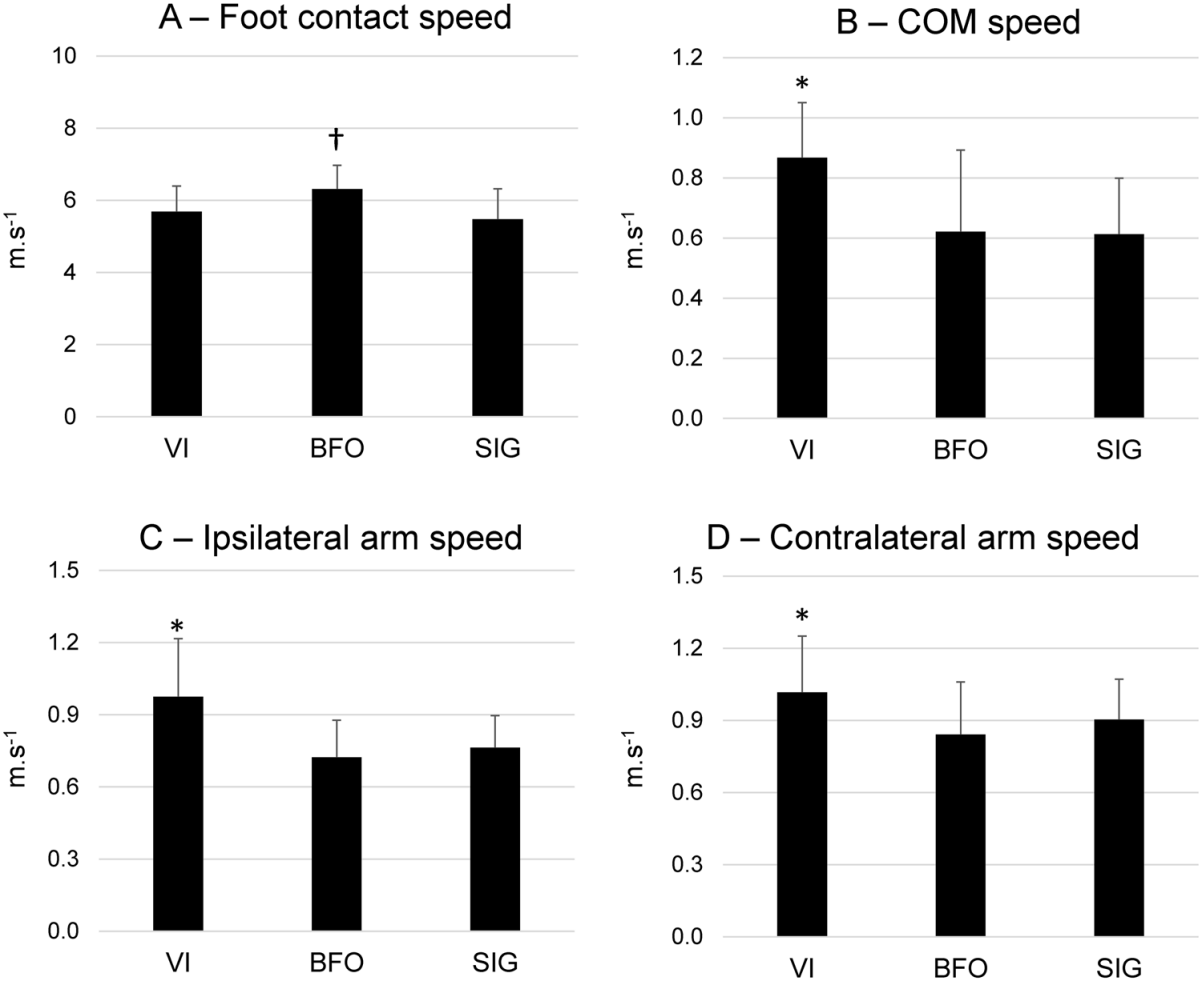
Mean(SD) kicking foot speed at the instant of contact to the ball (**A**), the center of mass (COM) speed 1 second after passing (**B**), ipsilateral (**C**) and contralateral (**D**) arm speed from −250 to 250 ms around passing. Data for each subject were averaged across all passes performed for 1 minute for visually impaired individuals (VI), sighted blindfolded (BFO) and sighted individuals (SIG). *Denotes significant differences in relation to BFO and SIG (p < 0.05); ^†^denotes a significant difference in relation to VI and SIG (p < 0.05).

The SIG group performed the greatest number of passes (55 ± 7 passes) when compared to BFO and VI (7 ± 1 and 17 ± 7 passes respectively, p = 0.00001, ŋp^2^ = 0.94). The BFO group presented the fastest speed during foot contact to the ball (p = 0.035, ŋp^2^ = 0.29), whereas VI and SIG were similar (Fig. 3A). Regarding whole-body movements, the VI group demonstrated the faster COM speed after passing (Fig. 3B, p = 0.01, ŋp^2^ = 0.23), as well as the fastest ipsilateral (Fig. 3C, p = 0.012, ŋp^2^ = 0.30) and contralateral arm speed (Fig. 3D, p = 0.036, ŋp^2^ = 0.12).

### Trunk kinematics during passes

**Figure 4:**
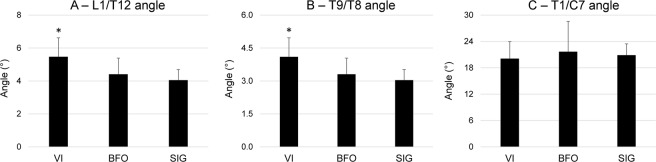
Mean(SD) flexion angle at the L1/T12 level (**A**), T8/T9 level (**B**) and T1/C7 level (**C)**. Data for each subject were averaged across all passes performed for 1 minute for visually impaired individuals (VI), sighted blindfolded (BFO) and sighted individuals (SIG). *Denotes significant differences in relation to BFO and SIG (p < 0.05).

The trunk flexion angle at the lumbar (L1/T12, Fig. 4A) and thoracic levels (Fig. 4B) were significantly greater for VI in comparison to both BFO and SIG (p = 0.045, ŋp^2^ = 0.31). No significant changes were found for the trunk flexion angle at the cervical level (Fig. 4C).

## Discussion

Here we tested for the first time the differences in movement patterns of VI players compared to sighted players with and without visual feedback. The main results from the kinematic analysis were that VI players reach slightly slower maximum speed and turning speed compared to sighted players in the shuttle test while performing a greater number of strides to cover the same distance. However, the BFO group was the slowest and presented a substantial increase in the number of strides to complete the task. Regarding ball passes, VI players hit the ball with similar speed compared to the SIG group, but they increase arms movement speed during passes. Moreover, VI players present greater COM speed, concomitant to increased trunk flexion, after passing. Increased trunk flexion after passing was also found for the BFO group, which seems an immediate adaptation to the lack of visual contribution to performing such a movement pattern. The results from this study can substantially contribute to increasing the understanding of the biomechanical demands of sports performance in blind athletes, potentially assisting coaches and product developers to adapt training procedures and equipment.

### Maximal and turning speed during 5-m shuttle run test

In walking, individuals with a visual impairment show adaptation strategies towards a more cautious pattern, as they seem to depend more on tactile feedback information from the foot’s plantar surface^18^. In is also known that congenitally blind children tend to take shorter strides, walke slower, and spend more time in the support phase of the gait than sighted children^19^. However, results on adults are unclear, as visually impaired adults manage to maintain a similar^20^ or inferior^21^, or superior^22^ walking speed than sighted blindfolded adults. Some controversies in the literature may be related to different experimental protocols, as the work of Gori and co-workers involved two-dimensional shape reproduction following a moving sound. Our results regarding maximum and turning speed during running suggested that VI players ran approximately 30% slower when compared to SIG. However, the BFO group presented the lowest maximum and turning speed across groups, due to the lack of long-term adaptations to running blindfolded.

Blindfolded football players presented a shorter stride length, which consequently reduces running speed. Furthermore, arm movements may be a key contributor to postural maintenance during ambulation of VI individuals. In fact, it has been shown that young VI individuals run slower than sighted individuals^19^. These VI individuals ran using shorter stride length and lower range of motion of the hip joint when compared to sighted individuals. They also kept stride contact longer and were airborne for a shorter time than the other peers^23^. Blindfolded sighted people may present even greater motor adaptations in their gait patterns, as walking without visual feedback information is a novel situation. This observation points towards an important role for multisensory integration during development, whereby the other sensory modalities are able to, at least partially, take over the role of visual information in the control of walking.

### Ball passing performance and postural adjustments

As expected, SIG performed a greater number of passes against a wall compared to VI players, but sighted participants performed BFO had a reduction of 86 ± 2% in their passing performance, performing less than 50% of what VI players could achieve. Foot speed during penalty kicking can range from 13 to 21 m.s^−1^ in youth players^7^, but no literature has been found describing foot speed during ball passing, in which our participants presented foot speed ranging from 4 to 8 m/s^−1^. Moreover, the BFO group presented greater foot speed while contacting the ball, which may indicate a lack of proper control to perform the passes compared to SIG and VI. Regarding posture, VI players presented greater arm movement speed to perform the passes. Previous studies have shown that arm movements are important to maintain and optimize postural control and reduce risks of falls^24^ which may be an additional strategy to improve balance control under restricted vision conditions.

The VI presented greater trunk flexion at L1/T12 and T9/T8 spine segments when compared to sighted individuals while performing passes. Vision is confined to frontal space, and mostly at head level in humans and most animals^25,26^. In the lower space actions are mediated by foot, and during ambulation, audio and motor feedback are linked. The representation of auditory frontal space around the chest is more accurate than the auditory frontal representation around the foot^27^. Therefore, forward leaning of the trunk seems to be a strategy related to maximizing the quality of auditory inputs to guide postural control during passing/receiving the ball. Interestingly, there was also a trend for BFO individuals to lean the trunk forward during passes. There were no instructions on how sighted participants should behave while blindfolded, therefore this postural adaptation seems an immediate strategy from the CNS to cope with the lack of visual inputs when spatial orientation is needed. Our data provide the first insights on the performance of VI players and can contribute to assisting coaches and product developers to adapt training procedures and equipment.

### Limitations

The limitations to the present study are (1) the limited number of football players. In Italy this kind of football is still at an amatorial stage, played mainly in spare time. This makes difficult to organize experimental settings with larger patient populations. As a consequence, the low number of participants limits the generalization of the findings; (2) Sighted participants were blindfolded and received a familiarization period in such condition. Therefore, a learning effect might have occurred during the BFO condition. This learning effect may be beneficial for the study design, as sighted players had to accommodate their sensory strategies to the novel vision-restricted condition. Furthermore, some of the results, such as the BFO forward trunk leaning during passes, indicated that BFO performance was changed towards the VI performance. This result is an indication that VI players may present the most effective adaptations to perform such motor tasks (3) The use of inertial motion capture for describing trunk flexion/extension may present limitations. There is an acceptable accuracy of inertial motion capture systems to estimate trunk flexion/extension angles^28^, but results must be considered protocol specific. Finally, the lack of validation tests for the ball passes on visually impaired players is a limitation. Therefore, the results of this test must be interpreted with caution.

In summary, we found that visually impaired players presented slower running and turning speed when compared to sighted players, but sighted blindfolded participants were slower than the visually impaired players. The visually impaired players hit the ball with similar speed compared to the SIG group, but they increase arms movement speed during passes, likely to maximize postural stability. Moreover, visually impaired players present greater center-of-mass speed, concomitant to increased trunk flexion at lumbar and thoracic levels, after passing. Such change in trunk position was also found in the blindfolded group, suggesting that leaning forward may be an immediate adaptation to the lack of visual contribution when targeting an object traveling in the opposite direction.

### Practical applications

These results can have some practical application. The first one is to provide to blind football players some indexes about how their football activity is performed compared to sighted players. This might be important for football trainers who are usually sighted to train the sport activity to reach these indexes. On the other hand, it can be also used to try to correct the motor behaviors that differ between sighted and blind players to verify if a more sighted like performance can optimize the results of the game. Starting from these results it would also possible to develop an application for football trainers and also for self-evaluation to quantify and train motor abilities of blind football players to reach optimal performances.

## Supplementary information

Supplementary Table 1
