# Supplementary material for: Kinematic Profile of Visually Impaired Football Players During Specific Sports Actions
Source: Sci Rep. 2019 Jul 23;9:10660. doi: 10.1038/s41598-019-47162-z (PMC6650599; doi:10.1038/s41598-019-47162-z)
Supplement: Supplementary file 1 — Supplementary Table 1 [file 41598_2019_47162_MOESM1_ESM.docx]

**KINEMATIC PROFILE OF VISUALLY IMPAIRED FOOTBALL PLAYERS DURING SPECIFIC SPORTS ACTIONS**

Sara Finocchietti^1^

Monica Gori^1^

Anderson Oliveira^2^

Supplementary Materials

**Supplementary Table 1**. Mean(SD) number of passes performed in 60 seconds by 11 young healthy and sighted recreational football players. The tests were performed in three different days, interspaced between 1-2 days. The intra-class correlation coefficient across these tests was 0.995.

| **Tree-day testing ball passes** | | | |
| --- | --- | --- | --- |
|  | **Day1** | **Day2** | **Day3** |
| **S1** | 55 | 55 | 57 |
| **S2** | 57 | 54 | 54 |
| **S3** | 59 | 61 | 58 |
| **S4** | 56 | 62 | 54 |
| **S5** | 56 | 57 | 52 |
| **S6** | 58 | 59 | 57 |
| **S7** | 51 | 50 | 51 |
| **S8** | 50 | 52 | 51 |
| **S9** | 60 | 61 | 59 |
| **S10** | 56 | 54 | 55 |
| **S11** | 51 | 51 | 53 |
| **Mean** | **55.36** | **56.00** | **54.64** |
| **SD** | **3.35** | **4.27** | **2.80** |
